# Patient level dataset to study the effect of COVID-19 in people with Multiple Sclerosis

**DOI:** 10.1038/s41597-024-02978-x

**Published:** 2024-01-31

**Authors:** Hamza Khan, Lotte Geys, Peer Baneke, Giancarlo Comi, Liesbet M. Peeters

**Affiliations:** 1 University MS Center (UMSC), Hasselt, Pelt Belgium; 2 UHasselt, Biomedical Research Institute (BIOMED), Agoralaan, 3590 Diepenbeek Belgium; 3 UHasselt, Data Science Institute (DSI), Agoralaan, 3590 Diepenbeek Belgium; 4 The D-Lab, Department of Precision Medicine, GROW-School for Oncology, Maastricht University, Maastricht, Netherlands; 5 Multiple Sclerosis International Federation, London, United Kingdom; 6 Department of Neurology, Scientific Institute S. Raffaele, via Olgettina 48, 20132 Milan, Italy

**Keywords:** Viral infection, Multiple sclerosis

## Abstract

Multiple Sclerosis (MS) is an inflammatory autoimmune disease of the central nervous system, causing increased vulnerability to infections and disability among young adults. Ever since the outbreak of coronavirus disease 2019 (COVID-19), caused by severe acute respiratory syndrome coronavirus 2 infections, there have been concerns among people with MS (PwMS) about the potential interactions between various disease-modifying therapies and COVID-19. The COVID-19 in MS Global Data Sharing Initiative (GDSI) was initiated in 2020 with the aim of addressing these concerns. This paper focuses on the anonymisation and publicly releasing of a GDSI sub-dataset, comprising data entered by PwMS and clinicians using a fast data entry tool. The dataset includes information on demographics, comorbidities and hospital stay and COVID-19 symptoms of PwMS. The dataset can be used to perform different statistical analyses to improve our understanding of COVID-19 in MS. Furthermore, this dataset can also be used within the context of educational activities to educate different stakeholders on the complex data science topics that were used within the GDSI.

## Background & Summary

Multiple Sclerosis (MS) is a chronic neuroinflammatory autoimmune disease of the central nervous system (CNS). In MS, nerve fibres in the CNS are affected, resulting in demyelination and axonal damage, leading to varying degrees of loss of functional capabilities^1^. MS is prevalent between the age group of 20–40 years and is more common in women than in men, with at least twice as many women affected. While the exact aetiology of MS remains unclear, an interplay between genetic predisposition and environmental factors could contribute to its observed prevalence^2–4^. Moreover, due to the combination of pathophysiology, treatment and natural history of MS, people with MS (PwMS) are four times more prone to contracting infections than the general population^5^.

Severe respiratory syndrome coronavirus-2 (SARS-CoV-2) is the virus responsible for the 2019 novel coronavirus disease (COVID-19), which has killed more than one million people worldwide^6^. Given the diverse spectrum of COVID-19 disease patterns, ranging from mild to severe^7,8^, and the reported immune deficiencies in PwMS^9^, there were concerns about whether taking immunosuppressive or immune modifying medications affects the course and outcome of COVID-19 in PwMS^10^. To address these concerns, a multi-stakeholder data-sharing initiative known as the COVID-19 and MS Global Data Sharing Initiative (GDSI) was set up. The GDSI was guided by the joint efforts of the Multiple Sclerosis International Federation (MSIF) (MSIF: https://www.msif.org/) and the Multiple Sclerosis Data Alliance (MSDA) (MSDA: http://msdataalliance.org), where they leveraged their expertise and network to enable various data partners to share their data in various ways, resulting in three different data streams. The main objective of the GDSI was to investigate the effect of immunosuppressants or immune-modifying medications on COVID-19 or its outcomes. Moreover, the initiative aimed to scale up the COVID-19 data collection efforts and provide the MS community with data-driven insights during the pandemic^10^.

First and foremost, the key areas of interest among completed and ongoing data collection efforts related to COVID-19 in MS were agreed upon. This was done via a global consensus-building teleconference, in which the variables related to COVID-19, COVID-19 severity, COVID-19 treatment, demographic information, MS history and severity, information on DMT use, comorbidities and certain lifestyle behaviours were identified as important to be included in the core dataset^10,11^.

Subsequently, the global MS community was engaged to share the documentation of the COVID-19 status of PwMS. MS registries and cohorts from different countries were included in the initiative, and they were asked to share their core dataset via a central platform provided by QMENTA (https://www.qmenta.com/). Additionally, via a fast data entry tool, PwMS and/or their clinicians could share data directly in the platform. The data was securely stored by QMENTA, which is a company that provided a cloud-based platform that facilitates the aggregation, standardisation, and visualisation of various types of data, particularly clinical and imaging data^10,11^.

### Rationale for Release and Limitations of the Dataset

This study is focused on publishing the data that was collected via the fast data entry tool. For the purposes of this paper, the data collected through this tool will be hereafter referred to as “direct entry dataset”. The release of the GDSI direct entry dataset^12^ is an important step towards the reproducibility and transparency of the research within the domain of MS. The processing and acquisition of this dataset^12^ have been performed keeping the European General Data Protection Regulation (GDPR) framework in mind^13^ and therefore for the first time, we are making this dataset^12^ publicly available.

As of the 31st of December 2022, the GDSI has been closed, meaning the central platform is no longer active, and all data available in the central platform has been deleted. The data originally provided by the participating data partners (via a data stream different from the direct entry) is kept securely by the individual data partners and can be retrieved if necessary for future (research) opportunities. The main motivation to share the direct entry dataset^12^ under an open license is for it to remain accessible for research beyond the GDSI. In this way, the availability of the direct entry dataset^12^ can aid in advancing our understanding of the effects of COVID-19 on PwMS. Moreover, it can serve as a validation dataset for different analytical models. It is important to note that the direct entry dataset^12^ differs from the datasets used in the previously published work^11^ because, besides being collected using the fast data entry tool, it has also been further anonymised using K-anonymity and ℓ-diversity. The motivation to do that was to ensure the privacy protection of the individuals while maintaining the dataset’s continued usability. Another reason to share the dataset^12^ under an open license is to appreciate and acknowledge the time invested by PwMS and clinicians that contributed to the GDSI direct entry dataset. Further information on the anonymity and acquisition of the data can be found in the methodology section.

The set-up, both from a technical- as well as governance perspective, that was used within the GDSI was unique. It allowed rapid data sharing at global scale and delivered data-driven insights to urgent clinical questions. The different data science concepts that were introduced and implemented within the GDSI could be leveraged to address other urgent questions in need of data-driven insights. However, a knowledge gap remains between the experts in MS (understanding the clinical questions) and the data scientists (understanding the technical architecture). There is a need for education to allow clinical experts to better understand complex data science-related topics. We are convinced that this openly licensed direct entry dataset^12^ can support this objective. The MS Data Alliance is a global non-for-profit multi-stakeholder organisation that aspires to overcome the socio-technical challenges that arise when scaling-up real-world MS data. One of the strategic objectives within the MS Data Alliance Academy is to educate the MS community on data science related topics through workshops, online tutorials and fellowship programs allowing personalised mentoring and training. The MS Data Alliance aspires to develop and host a series of workshops explaining how the GDSI was set-up. To allow the participants of these workshops to gain hands-on experience, the direct entry dataset^12^ described in this paper serves as the perfect resource to allow reproducing the GDSI set-up. Increased understanding of how the GDSI was set-up and how data-sharing at scale was facilitated will accelerate the collaborative research that is urgently required to deliver data-driven insights.

## Methods

The data for the GDSI was collected in three ways: (1) by individuals directly entering the data into a central platform via the fast data entry tool, (i.e., the direct entry dataset^12^) (2) by collecting de-identified data from participating MS registries and cohorts, which were regularly invited to upload their COVID-19 in MS core data, and (3) by acquiring aggregated results from certain registries that did not provide individual patient information. Since, the scope of this study only encompasses the direct entry dataset, the methodology section of this paper will discuss the data collection process, anonymization, and analysis methods pertaining specifically to the direct entry dataset. The details of the infrastructure of the overall GDSI for data collection and ethical approvals have been described previously^10,11^

In the context of the direct entry dataset^12^, our study followed the ethical guidelines and received approval from the ethics committee of Hasselt University to further anonymize and publish the data collected via the fast data entry tool in the public domain as long as the anonymity of the patients is fully respected (Comité voor Medische Ethiek UHasselt, CME2020/025 AMD3). After agreement upon the variables to be collected via the direct entry dataset^12^ as described in the previous section, a small cell risk assessment (SCRA) was performed upon the variables only by P-95 (P-95: https://www.p-95.com/). P-95 is a consulting company that was deemed a trusted third party by the ethical committee of Hasselt University and was tasked to minimise the risk of a patient’s identity disclosure. As a result, the variables were divided into three different categories: “direct identifiers”, “sensitive variables”, and “indirect identifiers”, also known as identifiers, sensitive attributes and non-sensitive or quasi-identifiers, respectively^14^. Direct identifiers are structurally unique variables that can directly identify a person without extra information such as social security number, full name etc. Sensitive variables refer to the variables that have content that a respondent would not like to disclose, such as religion, political alignment, COVID-19 diagnosis etc. Consequently, indirect identifiers are non-structurally unique variables that can re-identify an individual if combined with other indirect identifiers within or from another dataset^14^.

Following that, entering data via the fast data entry tool was made possible on a global level to two distinct categories of users, namely clinicians and PwMS. The outreach to these groups included direct emails to clinicians working in the field of MS, engaging with MS patient societies, dissemination of information via newsletters and promotion of the tool and the intended use of the data acquired by the tool on the MSDA, MSIF websites and social media platforms. The participation and sharing of data via either data stream was on voluntary basis.

Upon accessing to the direct entry tool, a questionnaire was given to the users where they had to fill in the details in line with the variables^10^ decided in the core dataset. Additionally, while asking for predetermined variables, the questionnaire did not ask for any directly identifiable personal data such as name, social security number, address, etc., thus leading to the platform’s inability to identify individuals that uploaded their own or their patient’s data via the fast data entry tool. The tool was available globally and since at the time COVID-19 was a novel disease, the extensive geographical coverage made sense to ensure the gain of a high volume of data to gain insights. The data regarding specific healthcare facilities however, of which the respective respondents were part of, was not collected. The tool has been taken down since the 3rd of February, 2022.

**Fig. 1:**
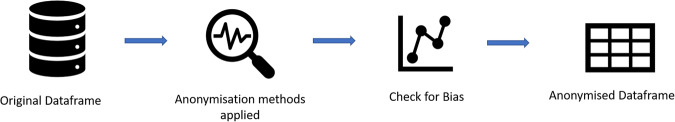
Anonymization Workflow for the Global Data Sharing Initiative Open Source Dataset. The workflow shows the anonymization process for the Global Data Sharing Initiative open source dataset. The dataset is first acquired, then aninymization methods are applied. The resulting anonymized dataset is then checked for bias using the chi-squared test independence. The final is an anonymized dataframe.

Once the acquisition of the direct entry dataset^12^ was finalised, steps were taken on the indirect identifiers towards applying further de-identification strategies such as data generalisation and data suppression. The former replaces a data value with a less precise value by applying binning, categorisation or rounding, while the latter involves the removal of the entire column. Subsequently, privacy-preserving techniques such as K-anonymity and ℓ-diversity were applied as well to prevent any possible re-identification attacks in the future (see Fig. 1).

**Fig. 2:**
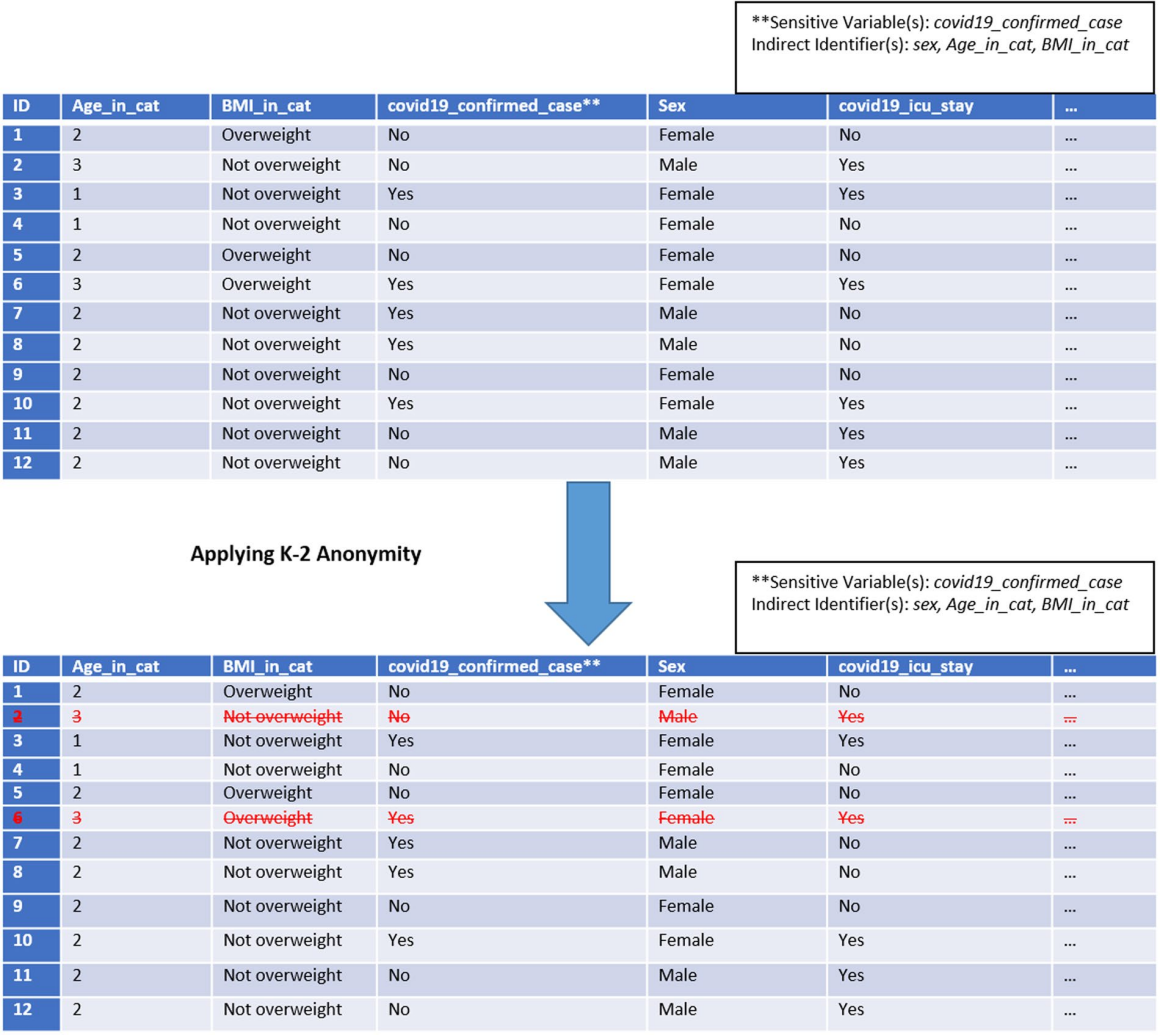
Anonymised Dataset with Redacted Rows. The mock dataset shown is K-2 anonymised using the K anonymity method, where each combination of indirect identifiers must occur at least two times. Rows that do not meet this criterion are indicated by crossed-out red font and subsequently removed.

K-anonymity is a privacy-preserving technique that aims to make the data in indirect identifiers indistinguishable. This effectively means that at least “K” individuals in the dataset should share the same set of attributes of indirect identifiers. A dataset is thus said to have achieved K-anonymity if all combinations of values for indirect identifiers in the dataset are present in at least K different records^15^. For example, Fig. 2 shows a dataset that has achieved a K-anonymity of 2. This is because the combination of indirect identifiers such as sex, Age_in_cat and BMI_in_cat occurs at least two times.

**Fig. 3:**
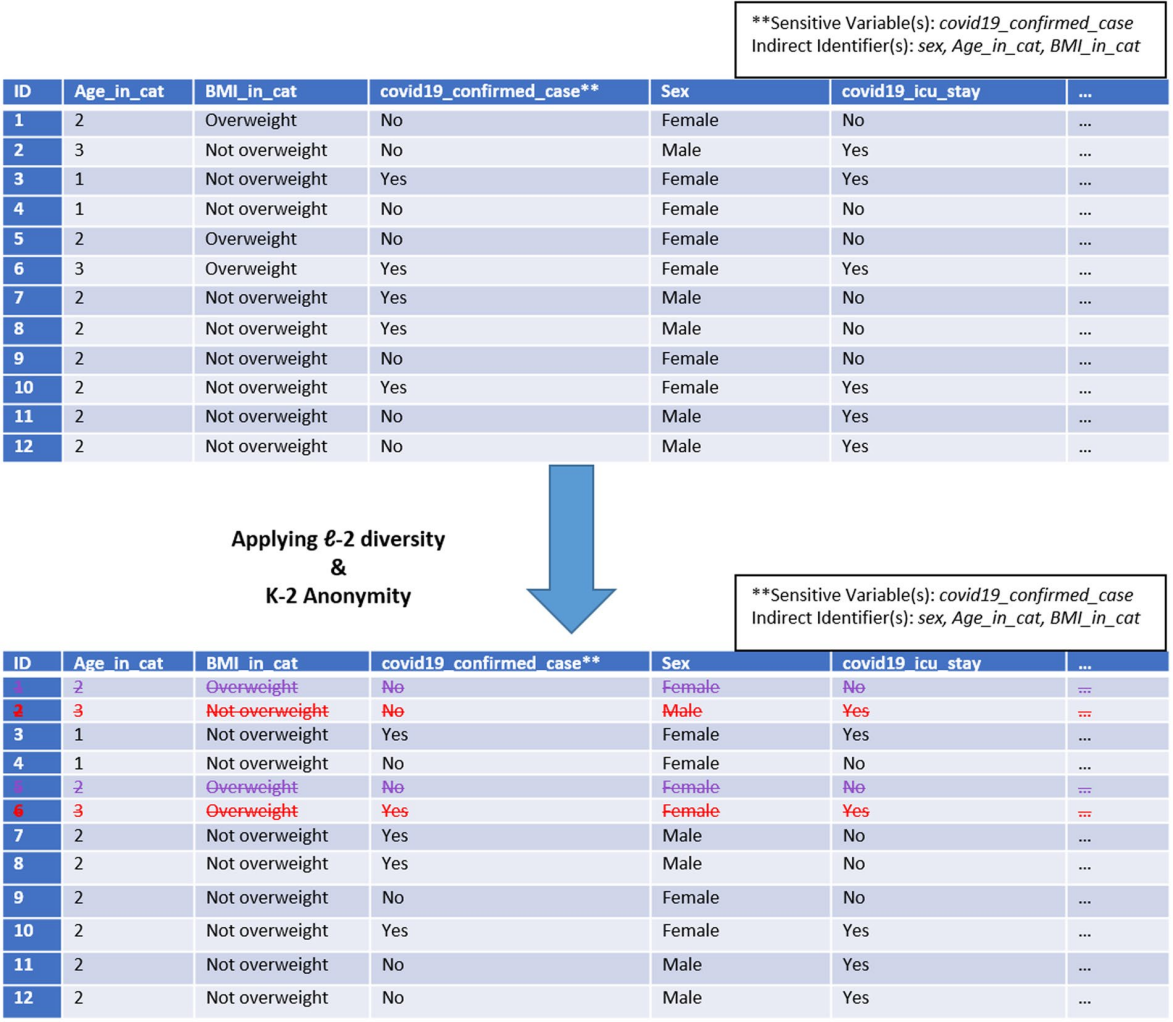
Anonymised and Diversified Dataset. The mock dataset shown is K-2 anonymised using the K anonymity method and l-2 diversified using the l-diversity technique. To ensure privacy, rows that do not meet the k-2 anonymity criterion are marked with a red crossed-out font, and rows that fail to satisfy the l-2 diversity requirement are market with a purple crossed-out font and subsequently eliminated. The dataset is required to have at least two occurrences of each combination of indirect identifiers and at least two classes in the sensitive variable column.

While K-anonymity can prove as an effective strategy for preventing identity disclosure, it nevertheless cannot ensure full protection of sensitive attributes, which can lead to semantic attacks, such as skewness and/or similarity attacks^16^. The ℓ-diversity model can potentially protect against these attacks. The ℓ-diversity model acts as a plugin on top of the K-anonymity model and focuses on diversity in sensitive attributes. ℓ-diversity is attained if each equivalence class of a K-anonymised dataset contains at least “ℓ” well-represented values for each sensitive attribute^17^. Figure 3 displays a dataset having K-anonymity and ℓ-diversity of 2. The dataset has at least two diverse values in the only sensitive variable, i.e., “covid19_confirmed_case”, for each combination of indirect identifiers. The rationale for choosing K-anonymity and ℓ-diversity stemmed from its previous use in literature^18^.

Upon the completion of the anonymisation of the dataset^12^, a bias assessment check was performed. Given the categorical composition of the indirect identifiers and sensitive variables, the relatively small sample size and the class imbalance in variables, a chi-squared test was conducted on the indirect identifiers and sensitive variables in the anonymised and the original dataset. Values of p < 0.05 are considered statistically significant. A SCRA was performed on the anonymised dataset^12^ by P-95 to ensure that the anonymised dataset is safe to be made public under an open licence. For analysis purposes, the anonymisation and statistical analysis of this dataset^12^ were performed using Python libraries such as matplotlib 3.6.0^19^, pandas 1.5.3^20^, NumPy 1.24^21^ and SciPy 1.0^22^. The interface used was Jupyter notebook 5.0^23^.

## Data Records

In this section, a more detailed description of the variables included in the accessible dataset has been provided, which is available at PhysioNet and can be accessed via 10.13026/77ta-1866. This dataset^12^ consists of 1141 PwMS.

In terms of demographical characteristics, as shown in Tables 1, 77.8% of the cohort were within the age range of 18–50 years, with a distribution of 79.22% females and 20.77% males. The majority of the patients (75.02%) had a BMI below 30, and a significant portion (43.38%) were current or former smokers.

**Table 1:** Demographical characteristics of the cohort.

No.	Variable	Percentage	Missing
1	Sex		0.0%
	Female	79.22% (n = 904)	
	Male	20.77% (n = 237)	
2	Age		0.0%
	18–50	77.38%	
	51–69	22.61%	
	≥70	0.00%	
3	BMI		0.0%
	<30	75.02%	
	≥30	2.97%	
4	Current or former smoker	43.38%	56.61%

Abbreviations: BMI = Body Mass Index.

The health status, as detailed in Tables 2, 5.2% of the cases had a “confirmed” COVID-19 diagnosis, while “not_suspected” or “suspected” cases comprised 75.19% and 19.54%, respectively. Hospitalisation occurred in 5.25% of patients, and ICU admission was necessary for 13.67%. Among the hospitalised patients, a small proportion, 0.43%, required artificial ventilation. The dataset^12^ also indicates that 24.8% of the cohort reported having comorbidities, with 79.4% having relapsing-remitting MS (RRMS) and 9.11% having primary progressive MS (PMS). Additionally, 45.83% had an EDSS score between 0–6.

**Table 2:** COVID-19 and MS diagnosis and severity, and comorbidities.

No.	Variable	Percentage	Missing
1	COVID-19 diagnosis		0.0%
	Confirmed COVID-19 cases	5.2% (n = 60)	
	Suspected or Non Suspected	94.73% (n = 1081)	
2	Hospitalisation		0.0%
	Yes	5.25%	
	No	94.74%	
3	ICU admission		0.0%
	Yes	13.67%	
	No	8.15%	
	Not applicable	78.17%	
4	Artificial Ventilation		0.43%
	No	99.21%	
	Yes	0.35%	
5	Has comorbidities		24.80%
	yes	28.74%	
	no	71.25%	
6	MS phenotype		0.0%
	RRMS	79.40%	
	PMS	9.11%	
	Other	11.48%	
7	EDSS 0–6	45.83% (n = 523)	54.16%

Abbreviations: COVID-19 = coronavirus disease 2019; ICU = Intensive Care Unit; MS = Multiple Sclerosis; RRMS = Relapsing Remitting Multiple Sclerosis; PMS = Progressive Multiple Sclerosis; EDSS = Expanded Disability Status Scale.

Table 3 outlines the medication and treatment details. The most commonly used disease-modifying therapy (DMT) was dimethyl fumarate, utilized by 12.7% of the patients, followed by fingolimod (10.16%) and natalizumab (5.69%). The dataset^12^ also has missing values with the EDSS score and current or former smokers, among others, having 54.16% and 56.61% missing values, respectively.

**Table 3:** Disease modifying therapy information.

No.	Variable	Percentage	Missing
1	Taking glucocorticoids		6.13%
	yes	4.20%	
	no	89.65%	
2	DMT		14.11%
	Currently not using any DMT	8.85%	
	Alemtuzumab	1.13%	
	Cladribine	3.06%	
	Dimethyl fumarate	12.70%	
	Fingolimod	10.16%	
	Glatiramer acetate	5.60%	
	Interferon beta	8.23%	
	Natalizumab	5.69%	
	Ocrelizumab	7.36%	
	Rituximab	1.31%	
	Teriflunomide	5.21%	
	Glatiramer	5.60%	
	Other DMT	16.21%	

Abbreviations: DMT = Disease Modifying Therapy.

### Variables Descriptions

- **secret_name:** A unique identifier for the patient. “P_” or “C_” in the beginning indicates patient-reported and clinician-reported outcomes, respectively.
- **report_source:** Indicates the source from which the data is acquired. The variable has two unique values: “clinicians” for clinician-reported and “patients” for patient-reported. Possible values: “Patients” (92.63%), “Clinicians” (7.36%).
- **age_in_cat:** Indicates age in categories. Missing values for this field: 0.00%.
  - 0: if the age range is between 0 and <18.
  - 1: if the age range is between 18 and ≤50.
  - 2: if the age range is between 51 and ≤70.
  - 3: if the age range is 71 or greater.

- **bmi_in_cat2:** This variable represents the body mass index (BMI) of the patient. BMI is a statistical index that can estimate body fat in people of any age by dividing a person’s weight in kilograms by the square of height in metres^24^. The unique values in bmi_in_cat2 are “not_overweight” (75.02%) and “overweight” (2.97%). The possible missing values are 21.99%.
  - “not_overweight”: if BMI ≤ 30 kg/m^2^.
  - “overweight”: if BMI > 30 kg/m^2^.

- **covid19_admission_hospital:** Indicates the hospital admission status of the patient as a result of COVID-19. Has two unique values: “Yes” (1.31%) indicates admission in the hospital, and “No” (98.78%) indicates no admission. 0.00% missing values.
- **covid19_confirmed_case:** Confirmed COVID-19 diagnosis of the patient. Has two unique values: “Yes” (5.25%) if the diagnosis is positive and “No” (94.74%) if it’s otherwise. 0.00% missing values.
- **covid19_diagnosis:** It shows the perceived COVID-19 diagnosis of the patient. It has three unique values: “not_suspected” (75.19%), “suspected” (19.54%) and “confirmed” (5.25%). 0.00% missing values. “confirmed” refers to the condition if covid_19_confirmed_case has a value of yes. “suspected” if the patient had a suspicion of having covid-19 and any covid_19 symptoms such as sore throat, shortness of breath, pneumonia, fatigue, pain, nasal congestion and chills. “not_suspected” was assigned in case there was an absence of symptoms, suspicion of having COVID-19 or a confirmed negative diagnosis.
- **covid19_has_symptoms:** Indicates the presence or absence of COVID-19 symptoms. Has two unique values: “Yes” (31.77%) if there are one or more symptoms associated with COVID-19, such as fever, dry cough, fatigue, pain, sore throat, shortness of breath, nasal congestion, loss of taste or smell and pneumonia. “No” (68.22%) if there are no symptoms. 0.71% possible missing values.
- **covid19_icu_stay:** Indicates whether the patient stayed in the intensive care unit (ICU) of the hospital as a result of COVID-19. “Yes” (0.35%) if the person stayed in the hospital intensive care unit (ICU) and “No” (99.38%) if the person did not stay in the ICU of the hospital. 0.26% possible missing values.
- **covid19_self_isolation:** Self-isolation status of the patient, advised either by the clinician or self-reported. “Yes” (39.87%) if the person self-isolated and “No” (58.8%) if the person did not self-isolate. Possible missing values are 1.31%.
- **covid19_sympt_chills:** Presence of chills as a COVID-19 symptom. “Yes” (8.85%) indicates the presence of chills as a symptom, and “No” (12.88%) indicates otherwise. 78.26% possible missing values.
- **covid19_sympt_dry_cough:** Presence of dry cough as a COVID-19 symptom. “Yes” (17.17%) if there are symptoms of dry cough and “No” (7.44%) if there are no symptoms of it. 75.37% possible missing values.
- **covid19_sympt_fatigue:** Presence of fatigue as a COVID-19 symptom. “Yes” (20.94%) if there are symptoms of fatigue and “No” (4.55%) if there are no symptoms of it. 74.49% possible missing values.
- **covid19_sympt_fever:** Presence of fever as a COVID-19 symptom. “Yes” (12.35%) if there are symptoms of fever and “No” (12.62%) if there are no symptoms of it. 75.02% possible missing values.
- **covid19_sympt_loss_smell_taste:** Presence of loss of smell and taste as a COVID-19 symptom. “Yes” (7.17%) if there are symptoms of loss of smell and taste and “No” (13.75%) if there are no symptoms of loss of smell and taste. 75.37% possible missing values.
- **covid19_sympt_nasal_congestion:** Presence of nasal congestion as a COVID-19 symptom. “Yes” (13.67%) if there are symptoms of nasal congestion and “No” (9.46%) if there are no symptoms of it. 76.86% possible missing values.
- **covid19_sympt_pain:** Presence of pain as a COVID-19 symptom. “Yes” (16.3%) if there are symptoms of pain and “No” (7.88%) if there are no symptoms of it. 75.81% possible missing values.
- **covid19_sympt_pneumonia:** Presence of pneumonia as a COVID-19 symptom. “Yes” (1.22%) if there are symptoms of pneumonia and “No” (18.75%) if there are no symptoms of it. 80.01% possible missing values.
- **covid19_sympt_shortness_breath:** Presence of shortness of breath as a COVID-19 symptom. “Yes” (9.02%) if there are symptoms of shortness of breath and “No” (13.40%) if there are no symptoms of it. 77.56% possible missing values.
- **covid19_sympt_sore_throat:** Presence of sore throat as a COVID-19 symptom. “Yes” (14.89%) if there are symptoms of sore throat and “No” (9.02%) if there are no symptoms of it. 76.07% possible missing values.
- **covid19_ventilation:** Indicates whether the patient used a ventilator unit for ventilation during their hospital stay. “Yes” (0.35%) if ventilation was used, and “No” (99.21%) indicates otherwise. 0.43% possible missing values.
- **current_dmt:** Indicates the status of the disease-modifying therapy (DMT) at the time of data entry of the patient. There are three unique values in the variable: “yes” (77.12%), “no” (8.85%) and “never_treated” (14.02%). 0.00% possible missing values. The values are measured as follows:
  - yes: if the person is currently on a DMT
  - no: if the person is not currently on a DMT
  - never_treated: if the person has never been on a DMT

- **dmt_glucocorticoid:** Describes the status of intake of glucocorticoid. “Yes” (4.2%) if the person is taking glucocorticoid, and “no” (89.65%) states otherwise. 6.13% possible missing values.
- **edss_in_cat2:** Indicates the category in which the Expanded Disability Status Scale (EDSS) lies. The EDSS is one of the most commonly used disability assessment tools^25^. The EDSS is an ordinal scale from 0 to 10 that runs in increments of 0.5, with 0 indicating normal neurological status and 10 indicating death due to MS. Even though it has a few shortcomings, such as low reliability and sensitivity to change, it is still one of the preferred scales^25,26^. The variables had two unique values: “1” (0.00%) and “0” (45.8%). 54.16% possible missing values. The values were calculated as follows:
  - 0: if the EDSS is between 0 and < = 6.
  - 1: if the EDSS is >6.

- **pregnancy:** Pregnancy status of the patient. “Yes” (0.43%) if the person is pregnant and “No” (74.75%) if the person is not. Possible missing values are 24.80%.
- **sex:** The biological sex of the patient. “Male” (20.77%) for males and “Female” (79.22%) for females. Possible missing values are 0.00%.
- **ms_type2:** The type of Multiple Sclerosis phenotype. This variable has three unique values: “relapsing_remitting” (79.4%), “progressive_MS” (9.11%) and “other” (11.48%). Possible missing values are 0.00%. The values were calculated as follows:
  - relapsing_remitting: if the type of MS is relapsing-remitting MS (RRMS)
  - progressive_MS: if the type of MS is secondary progressive MS (SPMS) or primary progressive MS (PPMS)
  - other: if the type of MS is a clinically isolated syndrome (CIS) or empty or “not_sure” in case the patient or clinician was not sure.

- **current_or_former_smoker:** Indicates the smoking status of the patient. “Yes” (43.38%) indicates whether a patient is a smoker and/or has been a former smoker. “No” (0.00%) indicates otherwise. Possible missing values are 0.00%.
- **dmt_type_overall:** Indicates the specific type of DMT the person was on during data entry. The unique values in the variable are: “Currently on another drug not listed” (16.21%), “Currently on dimethyl fumarate” (12.7%), “Currently on fingolimod” (10.16%), “Currently not using any DMT” (8.85%), “Currently on interferon” (8.23%), “Currently on ocrelizumab” (7.36%), “Currently on natalizumab” (5.69%), “Currently on glatiramer” (5.6%), “Currently on teriflunomide” (5.52%), “Currently on cladribine” (3.06%), “Currently on rituximab” (1.31%), “Currently on alemtuzumab” (1.13%). Possible missing values are 14.11%. The values were calculated as follows:
  - “No information on DMT use”: if there is no information in the present or past history of DMT use by the person.
  - “currently not using any DMT”: if the person is currently not using any DMT but has used DMT in the past or has not used DMT at all.
  - “currently on interferon”: if the current DMT of the person is on interferon.
  - “currently on glatiramer”: if the current DMT of the person is on glatiramer.
  - “currently on natalizumab”: if the current DMT of the person is on natalizumab.
  - “currently on fingolimod”: if the current DMT of the person is on fingolimod.
  - “currently on dimethyl fumarate”: if the current DMT of a person is on fumarate.
  - “currently on teriflunomide”: if the current DMT of the person is on teriflunomide.
  - “currently on alemtuzumab”: if the current DMT of the person is on alemtuzumab.
  - “currently on cladribine”: if the current DMT of the person is on cladribine.
  - “currently on siponimod”: if the current DMT of a person is on siponimod.
  - “currently on rituximab”: if the current DMT of the person is on rituximab.
  - “currently on ocrelizumab”: if the current DMT of the person is on ocrelizumab.
  - “currently on another drug not listed”: if the person is on DMT other than the above-listed ones.

- **duration_treatment_cat:** The duration of treatment of MS. The variable has two unique values: “0” (3.59%) and “1” (3.59%). Possible missing values are 80.54%. The unique values were calculated as follows:
  - 0: if the duration of treatment is less than 11 years.
  - 1: if the duration of treatment is 11 years or more.

- **stop_or_end_date_combined:** Date in dd/mm/yyyy format indicating the stopping of DMT. Possible missing values are 28.13%.
- **covid19_outcome_levels_2:** The outcome of COVID-19. The variable has three unique values: “0” (98.68%), “1” (0.87%), and “2” (0.43%). Possible missing values are 0.00%. The unique values were calculated as follows:
  - 0: If the person has COVID-19 but has not been hospitalised.
  - 1: The person has COVID-19 and has been hospitalised.
  - 2: The person has COVID-19, has been hospitalised, has been in the intensive care unit and/or was in a ventilation facility.
  - 3: The person died due to COVID-19 (not present in this dataset).

- **has_comorbidities:** Indicates whether the person has any comorbidities. “Yes” (28.74%) indicates that there are comorbidities, and “No” (71.25%) indicates otherwise. Possible missing values are 0.00%.
- **com_cardiovascular_disease:** Indicates the presence of cardiovascular comorbidities. “Yes” (1.13%) indicates that there are comorbidities, and “No” (22.08%) indicates otherwise. Possible missing values are 76.77%.
- **com_chronic_kidney_disease:** Indicates whether the person has any chronic kidney disease. “Yes” (0.35%) indicates that there are chronic kidney comorbidities, and “No” (22.61%) indicates otherwise. Possible missing values are 77.03%.
- **com_chronic_liver_disease:** Indicates whether the person has any chronic liver disease. “Yes” (0.87%) indicates that there are chronic liver comorbidities, and “No” (22.43%) indicates otherwise. Possible missing values are 76.68%.
- **com_diabetes:** Indicates whether the person has any diabetes. “Yes” (1.48%) indicates that there is diabetes, and “No” (21.99%) indicates otherwise. Possible missing values are 76.51%.
- **com_hypertension:** Indicates whether the person has hypertension. “Yes” (4.64%) indicates that there is hypertension, and “No” (19.63%) indicates otherwise. Possible missing values are 75.72%.
- **com_immunodeficiency:** Indicates whether the person has an immunodeficiency. “Yes” (2.54%) indicates that there is immunodeficiency, and “No” (20.42%) indicates otherwise. Possible missing values are 77.03%.
- **com_lung_disease:** Indicates whether the person has any lung disease. “Yes” (2.80%) indicates that there is a lung disease, and “No” (20.50%) indicates otherwise. Possible missing values are 76.68%.
- **com_malignancy:** Indicates whether the person has any malignancy. “Yes” (1.05%) indicates that there is malignancy, and “No” (21.91%) indicates otherwise. Possible missing values are 77.03%.
- **com_neurological_neuromuscular:** Indicates whether the person has any neurological and/or neuromuscular comorbidity. “Yes” (2.19%) indicates that there is neurological and/or neuromuscular comorbidity, and “No” (21.12%) indicates otherwise. Possible missing values are 76.68%.
- **comorbidities_other:** Indicates names of other comorbidities that the patient might have that are not mentioned in the column names. Possible missing values are 79.66%

## Technical Validation

The technical validation of this dataset^12^ was performed by adhering to a well-defined schema that facilitated the acquisition of data in a standardised manner. Furthermore, an automated data quality assessment pipeline was integrated during the data acquisition phase, which ensured the accuracy and reliability of the dataset^12^. For instance, constraints were placed on data entry, such as limiting the age range and only allowing ICU admission entries for people who were also documented as being admitted to the hospital.

The geographical data was suppressed during the de-identification process because including it would have compromised the level k-anonymity that we intended to achieve. Moreover, data generalisation was applied on the age variable to represent it in a categorical format. This helped in maintaining the anonymity of the dataset^12^ while retaining its utility. No direct identifiers were present in the dataset^12^. Additionally, the dataset^12^ contained the following sensitive attributes, i.e., “covid19_ventilation”, “com_cardiovascular_disease”, “com_chronic_liver_disease”, “com_chronic_kidney_disease”, “com_diabetes”, “com_hypertension”, “com_immunodeficiency”, “com_lung_disease”, “com_malignancy’, “com_neurological_neuromuscular” and two indirect identifiers such as “sex”, and “age_in_cat”. Based on the indirect identifiers and the sensitive attributes, the anonymised dataset^12^ achieved a K-anonymity of 60 and ℓ-diversity of 2.

After running the chi-square test (χ²) on the indirect identifiers and sensitive variables in the anonymised and the original dataset, the null hypothesis stating that there are no significant differences in the distributions was evaluated. The details of the analysis are displayed in Table 4. Initially, all the variables except “age_in_cat” exhibited a statistically significant change in distribution. This is because the “age_in_cat” in the original dataset had an extra category with 6 individuals. However, since anonymisation is a trade-off between privacy preservation and usefulness, and given the small sample size of the minor class in age_in_cat, that category was eventually removed. Therefore, when the adjusted “age_in_cat” with the same number of categories in both original and anonymised datasets^12^ were analysed, the p-value became insignificant, i.e., (χ²(1) = 0.0038, p = 0.95).

**Table 4:** Chi-Squared test results for the sensitive and indirect-identifiers of the anonymised dataset.

Variable	Degrees of freedom	Chi-squared statistic	P-value (α = 0.05)
Age_in_cat (after adjusting)	1	0.0038	0.95
Sex	1	0.0198	0.88
covid19_ventilation	1	0.0007	0.97
com_cardiovasuclar_disease	1	0.0128	0.90
com_chronic_liver_disease	1	0.0047	0.94
com_chronic_kidney_disease	1	0.0005	0.98
com_diabetes	1	0.0082	0.92
com_hypertension	1	0.0157	0.90
com_immunodeficiency	1	2.0895	0.99
com_lung_disease	1	0.0001	0.98
com_malignancy	1	0.0093	0.92
com_neurological_neuromascular	1	0.0013	0.97
ms_type	4	0.0066	0.99
BMI_in_cat	1	0.1455	0.70
covid19_diagnosis	4	0.0027	0.99

Abbreviations: Age_in_cat = age in categories; covid19 = coronavirus disease 2019; com = comorbidity; BMI _in_cat = Body Mass Index in categories; MS = Multiple Sclerosis.

Thus, the analysis in Table 4 implied an insignificant change in the distribution of the variables in the original and anonymised dataset^12^ with respect to the indirect identifiers and sensitive attributes. While the absence of an insignificant change in the distribution does not necessarily imply an absence of bias, it does provide preliminary support for the utility of the anonymised dataset for further research purposes. Nevertheless, for certain specific categories, such as hospitalisation and intensive care unit (ICU) admission, the anonymised dataset^12^ was not able to fully capture the extreme minority cases. This discrepancy can be seen in Table 5.

**Table 5:** Original vs Anonymised: Differences in severe cases.

Variable	Sex	Original Data frame (%)	Anonymised Data frame (%)
Hospital Admission	Male	2.74	0.42
	Female	1.97	1.55
ICU Admission	Male	0.27	0.00
	Female	0.39	0.44

Abbreviations: ICU = Intensive care unit.

## Usage Notes

Despite the potential benefits, there are certain limitations of this dataset^12^ as well. First, the dataset^12^ does not take into account false positive or false negative cases of COVID-19. Second, the size of this dataset^12^ is small and can thus impede the statistical power of the analysis performed on it, resulting in possible imprecise estimates of the association between MS and COVID-19. A striking example is the presence of only 5.2% (n = 60) PwMS in the dataset^12^ with confirmed COVID-19 diagnosis. This can limit the robustness of the dataset’s ability to provide reliable insights regarding the impact of COVID-19 on PwMS. Lastly, the dataset^12^ can have a potential selection bias. This pertains specifically to the cases reported by PwMS themselves as critically ill and deceased cases would most likely not be included in the dataset^12^. Furthermore, some of the rows were also eliminated during the application of privacy enhancement strategies such as K-anonymity and ℓ-diversity. These limitations should be taken into consideration when interpreting the results of any analysis conducted using this dataset^12^.

## Data Availability

The dataset^12^ acquisition pipeline was developed using Python programming language, and the data is provided in a CSV format, which makes it compatible with a variety of data analysis tools and software packages. The pipeline utilized Python libraries, including matplotlib 3.6.0, pandas 1.5.3, NumPy 1.24, and SciPy 1.0 for data aggregation, statistical analysis (e.g., bias checks using chi-squared test), and visualization. Jupyter Notebook 5.0 served as the interface for the pipeline. To assist the user community in both the collection and analysis of the data, the code and tools developed for this dataset are available through GitHub. Users can access the repository, which contains the Python scripts, at https://github.com/hky365/Global-Data-Sharing-Initiative-.git. This can help users to reproduce the analyses, adapt the code for their specific needs, and collaborate with other researchers.

## References

[1] Calabresi PA (2004). Diagnosis and management of multiple sclerosis. Am Fam Physician..

[2] Cree BAC (2014). Multiple sclerosis genetics. Handb Clin Neurol..

[3] Hatch MN, Schaumburg CS, Lane TE, Keirstead HS (2009). Endogenous remyelination is induced by transplant rejection in a viral model of multiple sclerosis. J Neuroimmunol..

[4] Vandebergh M, Degryse N, Dubois B, Goris A (2022). Environmental risk factors in multiple sclerosis: bridging Mendelian randomization and observational studies. J Neurol..

[5] Montgomery S, Hillert J, Bahmanyar S (2013). Hospital admission due to infections in multiple sclerosis patients. Eur J Neurol..

[6] Yadaw AS (2020). Clinical features of COVID-19 mortality: development and validation of a clinical prediction model. Lancet Digit Health..

[7] Guan W (2020). Clinical characteristics of coronavirus disease 2019 in China. New England Journal of Medicine..

[8] Richardson S (2020). Presenting characteristics, comorbidities, and outcomes among 5700 patients hospitalized with COVID-19 in the New York City area. JAMA..

[9] Lamoureux G (1981). A clinical and immunological study of the effects of transfer factor on multiple sclerosis patients. Clin Exp Immunol..

[10] Peeters LM (2020). COVID-19 in people with multiple sclerosis: A global data sharing initiative. Mult Scler..

[11] Simpson-Yap S (2021). Associations of disease-modifying therapies with COVID-19 severity in multiple sclerosis. Neurology..

[12] Khan H, Geys L, Baneke P, Comi G, Peeters L (2024). PhysioNet.

[13] European Commission. Regulation (EU) 2016/679 of the European Parliament and of the Council of 27 April 2016 on the protection of natural persons with regard to the processing of personal data and on the free movement of such data, and repealing Directive 95/46/EC (General Data Protection Regulation) (Text with EEA relevance). *OJ*. (2016).

[14] The OECD Glossary of Statistical Terms. https://stats.oecd.org/glossary/.

[15] Sweeney L (2002). Achieving k-anonymity privacy protection using generalization and suppression. International Journal of Uncertainty, Fuzziness and Knowledge-Based Systems..

[16] Su B (2023). K-Anonymity privacy protection algorithm for multi-dimensional data against skewness and similarity attacks. Sensors (Basel)..

[17] Machanavajjhala A, Kifer D, Gehrke J, Venkitasubramaniam M (2007). L-diversity: Privacy beyond k-anonymity. ACM Trans. Knowl. Discov. Data..

[18] Yperman J, Popescu V, Van Wijmeersch B, Becker T, Peeters LM (2022). Motor evoked potentials for multiple sclerosis, a multiyear follow-up dataset. Sci Data.

[19] Hunter JD (2007). Matplotlib: A 2D graphics environment. Computing in Science & Engineering..

[20] team, T. pandas development. pandas-dev/pandas: Pandas (v1.5.3), 10.5281/ZENODO.7549438 (2023).

[21] Harris, C. Array programming with NumPy. 10.1038/s41586-020-2649-2 PMC7759461 32939066

[22] Virtanen P (2020). SciPy 1.0: Fundamental algorithms for scientific computing in Python. Nat Methods.

[23] Kluyver, T. *et al*. Jupyter Notebooks – a publishing format for reproducible computational workflows. in (eds. Loizides, F. & Scmidt, B.) 87–90, 10.3233/978-1-61499-649-1-87 (IOS Press, 2016).

[24] Nuttall FQ (2015). Body mass index. Nutr Today..

[25] Kurtzke JF (1983). Rating neurologic impairment in multiple sclerosis: an expanded disability status scale (EDSS). Neurology..

[26] Meyer-Moock S, Feng Y-S, Maeurer M, Dippel F-W, Kohlmann T (2014). Systematic literature review and validity evaluation of the expanded disability status scale (EDSS) and the multiple sclerosis functional composite (MSFC) in patients with multiple sclerosis. BMC Neurol..

